# Hybrid and adaptive control of functional electrical stimulation to correct hemiplegic gait for patients after stroke

**DOI:** 10.3389/fbioe.2023.1246014

**Published:** 2023-08-07

**Authors:** Yiqun Dong, Kangling Wang, Ruxin He, Kai Zheng, Xiaohong Wang, Guozhi Huang, Rong Song

**Affiliations:** ^1^ School of Biomedical Engineering, Shenzhen Campus of Sun Yat-sen University, Shenzhen, China; ^2^ The Key Laboratory of Sensing Technology and Biomedical Instrument of Guangdong Province, School of Biomedical Engineering, Sun Yat-sen University, Guangzhou, China; ^3^ Department of Rehabilitation Medicine, Zhujiang Hospital, Southern Medical University, Guangzhou, Guangdong, China; ^4^ School of Rehabilitation Medicine, Southern Medical University, Guangzhou, China; ^5^ Shenzhen Research Institute of Sun Yat-sen University, Shenzhen, China

**Keywords:** functional electrical stimulation, hemiplegic gait, stroke, iterative learning control, hybrid

## Abstract

**Introduction:** Gait, as a fundamental human movement, necessitates the coordination of muscles across swing and stance phases. Functional electrical stimulation (FES) of the tibialis anterior (TA) has been widely applied to foot drop correction for patients with post-stroke during the swing phase. Although the gastrocnemius (GAS) during the stance phase is also affected, the Functional electrical stimulation of the gastrocnemius received less attention.

**Methods:** To address this limitation, a timing- and intensity-adaptive Functional electrical stimulation control strategy was developed for both the TA and GAS. Each channel incorporates a speed-adaptive (SA) module to control stimulation timing and an iterative learning control (ILC) module to regulate the stimulation intensity. These modules rely on real-time kinematic or kinetic data during the swing or stance phase, respectively. The orthotic effects of the system were evaluated on eight patients with post-stroke foot drop. Gait kinematics and kinetics were assessed under three conditions: no stimulation (NS), Functional electrical stimulation to the ankle dorsiflexor tibialis anterior (SA-ILC DS) and FES to the tibialis anterior and the ankle plantarflexor gastrocnemius (SA-ILC DPS).

**Results:** The ankle plantarflexion angle, the knee flexion angle, and the anterior ground reaction force (AGRF) in the SA-ILC DPS condition were significantly larger than those in the NS and SA-ILC DS conditions (*p* < 0.05). The maximum dorsiflexion angle during the swing phase in the SA-ILC DPS condition was similar to that in the SA-ILC DS condition, with both being significantly larger than the angle observed in the NS condition (*p* < 0.05). Furthermore, the angle error and force error relative to the set targets were minimized in the SA-ILC DPS condition.

**Discussion:** The observed improvements can be ascribed to the appropriate stimulation timing and intensity provided by the SA-ILC DPS strategy. This study demonstrates that the hybrid and adaptive control strategy of functional electrical stimulation system offers a significant orthotic effect, and has considerable potential for future clinical application.

## 1 Introduction

Stroke ranks as the second leading cause of death globally, and one of the primary causes of disability, affecting 15 million people annually. From 1990 to 2019, stroke incidence increased by 70%, and the burden of stroke grew substantially (Melo et al., 2015). Stroke is a neurological disorder that leads to movement disorders, such as hemiplegic gait (Lyons et al., 2002; Johnson et al., 2016). Hemiplegic gait typically involves two distinct forms (Lee et al., 2014). On one hand, paralysis or significant weakness of ankle dorsiflexion muscle results in inadequate dorsiflexion, preventing the ability to lift the toes off the ground during the swing phase of gait (Prenton et al., 2016). On the other hand, weakness or spasticity of the plantarflexors hinders patients’ inability to both support their own weight and provide adequate forward propulsive force during the stance phase (Neptune et al., 2001). Consequently, identifying appropriate intervention strategies to address foot drop symptom and enhance forward propulsion is vital.

Functional electrical stimulation (FES) is the most common intervention for correcting foot drop (Gil-Castillo et al., 2020). A typical FES system integrates a stimulation unit, a network of sensors, and a controller that adjusts the output current based on information from the sensing network to stimulate muscles, helping patients regain muscle control and reshape the nervous system (Melo et al., 2015). FES was first applied to foot drop correction by Liberson et al. (Liberson et al., 1961), who applied electrical stimulation to the tibialis anterior (TA) muscle during the heel-off and heel-strike moment. Since then, numerous studies have investigated the control and effects of FES. However, most studies of FES have focused on single-channel stimulation of the TA using open-loop controllers (Cameron, 2010; Stein et al., 2010; Gil-Castillo et al., 2020). The stimulation parameters of these studies are generally preset and fixed, lacking feedback from gait information (Lyons et al., 2002; Kesar et al., 2009). This traditional approach results in insufficient robustness to nonlinear, time-varying, and coupled responses of the system to the stimulated muscles. It also causes the rapid muscle fatigue of patients (Lynch and Popovic, 2008), which affects the ankle plantarflexion angle (Lee et al., 2014; Spaich et al., 2014). However, closed-loop control, another FES control method, can adapt stimulation parameters in real time to track predetermined targets (Nekoukar and Erfanian, 2012; Melo et al., 2015), adjusting the patients’ gait to emulate that of healthy individuals (van der Linden and Mercer, 2017) and perform natural physiological movements (Valtin et al., 2014). Closed-loop control strategies include finite state control (Hausdorff and Ring, 2008), artificial neural network (Riess and Abbas, 2001), proportional-integral-derivative control (Rouhani et al., 2017), fuzzy network (Jonic et al., 1999), and iterative learning control (ILC) (Seel et al., 2014; Valtin et al., 2014; Seel et al., 2015; Seel et al., 2016b). In particular, ILC algorithms have been found to address the convergence limitation, providing stable tracking performance as well as updating parameters to compensate for both internal and external disturbances (Arimoto et al., 1984; Freeman et al., 2012; Müller et al., 2017).

Furthermore, while traditional single-channel FES delivered to the TA increased ankle dorsiflexion angles during the swing phase, other gait deficits remained unaddressed. For instance, these deficits include decreased ankle plantarflexion, reduced swing-phase knee flexion, and diminished stance-phase propulsive force (Kesar et al., 2010). These deficits are mainly caused by the impaired ankle plantarflexor muscle, the gastrocnemius (GAS) (Chen et al., 2018a; Jiang et al., 2020). Therefore, applying hybrid control strategy of FES to mimic normal muscle activity in both TA and GAS should be helpful to the patients’ muscle acting in concert to improve gait symmetry and stability (Yang et al., 2012). Indeed, recent advancements in hybrid control strategy of FES have demonstrated promising orthotic effects (van Swigchem et al., 2010; Khamis et al., 2015; van Bloemendaal et al., 2016) and positive contributions to combating muscle fatigue (Nekoukar and Erfanian, 2012; Melo et al., 2015). However, significant challenges remain due to its complex combination with closed-loop control (Lynch and Popovic, 2008; Gil-Castillo et al., 2020). The dual-channel FES proposed by Kesar et al. (Kesar et al., 2009) provided patients with greater ankle plantarflexion angle, knee flexion angle and anterior ground reaction force (AGRF). Nevertheless, the timing of the stimulation of the two muscles obtained through a footswitch open-loop controller, may be unsuitable. In comparison to the single-channel case, it resulted decreased ankle dorsiflexion angles. In addition, the dual-channel FES designed by Lee et al. (Lee et al., 2014) improved both knee flexion angle and ankle dorsiflexion angle during the swing phase, but not ankle plantarflexion angle and AGRF, which may be caused by a simplified single switch system and the fixed stimulation intensity. In our previous studies, Chen and Jiang et al. (Chen et al., 2018a; Jiang et al., 2020) established a linear model based on walking speed to modulate stimulation timing, and used an ILC algorithm to adjust the stimulation intensity, improving the walking ability of patients with post-stroke foot drop. These previous studies have proven the benefit of integrating a speed-adaptive model and ILC algorithm in a single-channel FES control architecture to the TA (Chen et al., 2018a; Jiang et al., 2020). Therefore, a hybrid and adaptive control strategy that simultaneously regulates the stimulation timing and intensity is essential to enhance the orthotic effect of FES-assisted gait. Currently, such a strategy has not yet been realized.

The objective of this study is to establish a hybrid and adaptive control strategy of FES system, aiming at correcting hemiplegic gait physiologically and effectively, addressing foot drop and inadequate forward propulsion. To assess the orthotic effect of this system, the kinematic and kinetic data of patients after stroke were compared under conditions of no FES, single-channel FES, and the FES with hybrid and adaptive control strategy.

## 2 Methods

### 2.1 Subjects

Eight post-stroke subjects (seven males and one female, Table1) were asked to enroll in the study. Before the experiment, specialists conducted the Fugl-Meyer motor assessment of lower extremity (FMA-L). The inclusion criteria stipulated that the subjects must have experienced a singular stroke at least 6 months prior to the study, with the FMA-L score >20; possess the capability to walk independently for a minimum of 2 min continuously on a treadmill; and have sufficient passive ankle dorsiflexion range to achieve a neutral ankle angle (0°) or at least 5° of plantarflexion with the knee bent at 90°(Kesar et al., 2009). Before taking part in the experiment, all participants signed written informed consent, which was approved by the ethics committee of Zhujiang Hospital, Southern Medical University.

**TABLE 1:** Demographic and clinical information about subjects with stroke.

NO.	Sex	Age	Months after stroke	Lesion side	FMA-L	Comfortable speed (km/h)
1	M	62	23	R	24/34	2.0
2	M	53	15	R	27/34	1.0
3	M	66	7	L	24/34	0.8
4	M	67	32	L	25/34	1.3
5	M	58	15	R	27/34	2.4
6	M	24	17	L	24/34	0.8
7	M	59	19	L	24/34	0.9
8	F	22	6	R	26/34	0.8

Abbreviations: M, male, F, female, L, left, R:right, FMA -L, Fugl-meyer motor assessment for lower limb.

### 2.2 Experimental protocol

Prior to the start of the experiment, participants were requested to walk on a treadmill while ensuring safety by holding the handrail (Kesar et al., 2010). The purpose was to determine their most comfortable walking speed. To eliminate the impact of treadmill acceleration and deceleration on walking, each subject took at least 20 steps at their preferred speed (Gabel and Brand, 1994). Ankle and knee angles were measured in the sagittal plane when the subject was standing, and defined as the neutral angles, respectively. The neutral ankle angle was set at 0° (Wu et al., 2005). Positive ankle angle values indicated dorsiflexion, while negative values indicated plantarflexion.

The maximum stimulation intensity of the TA was ascertained either by the subject’s maximum tolerance or when the paralyzed ankle achieved 4.9° dorsiflexion while sitting, whichever occurred early, a customary approach in many previous studies (Chen et al., 2009; Kesar et al., 2010; Seel et al., 2016a). During the experiment, the actual stimulation intensity did not surpass the maximum threshold. The minimum stimulation intensity of the TA was set when the subject felt a slight stimulus, with the actual stimulation intensity being stronger than the minimum. For the GAS, the maximum stimulation intensity was determined when the subject reached maximum tolerance or when the subject stood in a position resembling terminal double support during gait, aiding in raising the paralyzed heel off the groud, whichever happened first (Kesar et al., 2009). The minimum stimulation intensity of the GAS was set at a level where the subject’s gastrocnemius muscle felt a slight stimulus. The actual stimulation intensity of the GAS during the experiment ranged between the maximum and minimum values.

During the experiment, subjects walked under the three distinct conditions: walking with no stimulation (NS), walking with single-channel FES delivered to the TA (SA-ILC DS), and walking with FES delivered to both the TA and the GAS (SA-ILC DPS). Subjects with post-stroke performed three trials for each condition in a random order, totaling nine trials per subject. During each trial, participants were instructed to walk at their most comfortable speed for a minimum of 2 min, with a 2-min rest interval between successive trials to avert muscle fatigue. The stimulation profile used in all conditions was biphasic waveform. The stimulation pulse width was set at 390 µs, and the frequency was 40 Hz.

### 2.3 System structure

**FIGURE 1:**
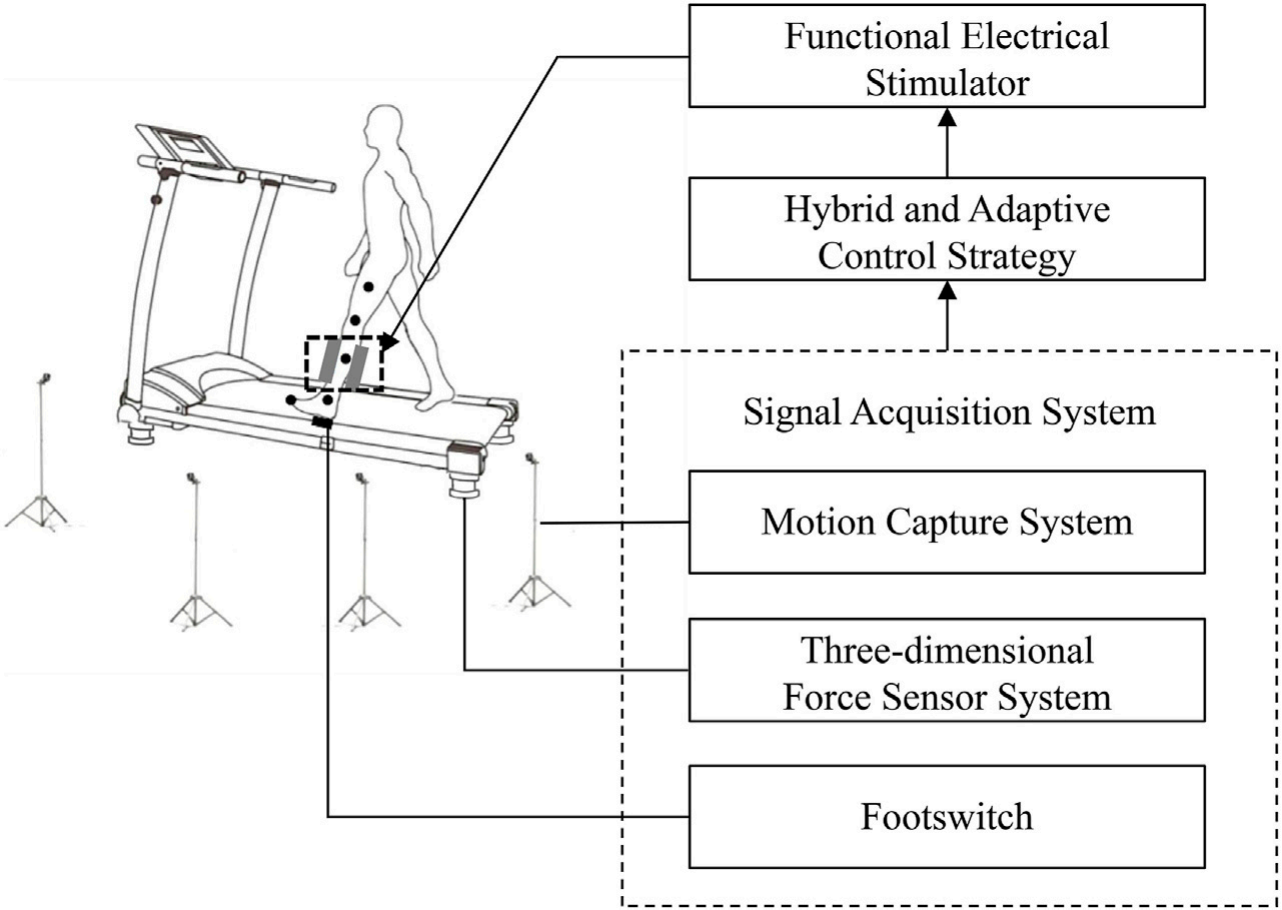
The structure of the FES system.

Figure 1 shows the structure of the FES system. The FES system included a treadmill (Y-P260EA, Yusheng, China) with four three-dimensional force sensors (CL-TR2, Obatel Automation Equipment, China) mounted underneath, a motion capture system (OptiTrack, Natural Point, United States of America), an A-D converter with 32-channel (USB-6343, National Instruments, Texas, United States of America), a footswitch (B-201, Tekscan, United States of America) and a functional electrical stimulator (P2-9632, Fisco, China). The three-dimensional force sensor system detected the kinetic signal of the subject walking on the treadmill at a sampling rate of 1000 Hz. The calibration method was based on the study by Belli et al. (Belli et al., 2001): horizontal forces ranging from 0 to 20 kg were applied on the treadmill using standard test weights. A linear relationship between the standard force and the actual measured force was observed. Linear fitting was performed, achieving a fitting accuracy greater than 0.99. The footswitch, which was positioned on the subject’s hind foot, detected heel-strike and heel-off events. The motion capture system acquired and stored kinematic data using four infrared cameras, capturing information at a frequency of 100 Hz. To facilitate this, the subject affixed five 12-mm spherical reflective markers to the paralyzed lower limb. The five markers used to measure knee and ankle angles were placed in a bottom-to-top sequence, following specific anatomical reference locations: the space between the second and third metatarsal bones, the lateral malleolus, the midshank, the lateral knee joint, and the mid-thigh (Kadaba et al., 1990). The aforementioned signals were transmitted to the PC via the A-D converter.

### 2.4 Control strategy

**FIGURE 2:**
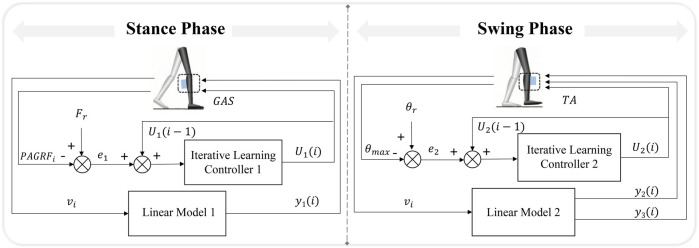
The hybrid and adaptive control strategy of the FES system.

The hybrid timing- and intensity-adaptive control strategy of FES system was applied to facilitate proper ankle movement during walking. The control strategy comprised two independent components: the stimulation timing was regulated by linear models, and the stimulation intensity was controlled by iterative learning controllers. The hybrid and adaptive control strategy of FES system is shown in Figure 2.

Linear models correlating walking speed with muscle activation phase parameters were established (Murley et al., 2014; Ma et al., 2015) due to the different activation of muscles at different speeds (Kadaba et al., 1990). The walking speed was calculated based on averaging the horizontal displacement change of the toe marker over the last five consecutive steps during gait (Chen et al., 2013). It should be noted that the study did not take into account the relative motion between the treadmill and the subjects’ center of mass. The activation timing parameters were composed of delay time $y_{1}\left(i\right)$ , $y_{2}\left(i\right)$ and duration time $y_{3}\left(i\right)$ . The calculation formulas were as follows:

$$y_{k}\left(i\right)=\alpha_{k}v_{i}+\beta_{k}$$ (1)

where k is the order of the outputs of the linear models, the coefficients $\alpha_{1}=-286.8$ , $\beta_{1}=541.6$ , $\alpha_{2}=-111.7$ , $\beta_{2}=416.9$ , $\alpha_{3}=-213.2$ , and $\beta_{3}=877.7$ . When the footswitch discovered the heel-strike event in the step i th, the stimulation for the GAS was initiated after the linear speed-adaptive time interval $y_{1}\left(i\right)$ , and the stimulation ended when the motion capture system detected that the toe-off event (Chen et al., 2018a; Jiang et al., 2020). The stimulation for the TA was triggered after the speed-adaptive time interval $y_{2}\left(i\right)$ when the footswitch found the heel-off event in the step i th, and the stimulation terminated after another speed-adaptive duration time $y_{3}\left(i\right)$ (Chen et al., 2018a; Jiang et al., 2020). The unit for $v_{i}$ was in meters per second, and the units for the parameters $y_{1}\left(i\right)$ , $y_{2}\left(i\right)$ , and $y_{3}\left(i\right)$ were seconds.

The rules for the iterative learning controllers to regulate the stimulation intensity for the GAS (when k= 1) and the TA (when k= 2) were as follows:

$$U_{k}\left(i\right)=T_{k}\times \left(U_{k}\left(i-1\right)+L_{k}\times e_{k}\right)$$ (2)

$$T_{k}=\left\{\begin{array}{c} \underline{I_{k}},I<\underline{I_{k}};\\ I,\underline{I_{k}}<I<\bar{I_{k}};\\ \bar{I_{k}},I>\bar{I_{k}}; \end{array}\right.$$ (3)

The target value of the peak anterior ground reaction force $F_{r}$ during the stand phase was acquired by the following formula (Ray et al., 2018).

$$F_{r}=0.15\times v_{treadmill}+0.035$$ (4)

where, $e_{1}$ was the error between the actual peak anterior ground reaction force ${PAGRF}_{i}$ and the target value $F_{r}$ during the stance phase. The target swing-phase ankle dorsiflexion angle was defined as 4.9°, which represents the maximum dorsiflexion angle observed in healthy individuals during walking (Arifin et al., 2003). The parameter $e_{2}$ represented the error between the actual maximum ankle dorsiflexion angle $\theta_{\max}$ and the target angle $\theta_{r}$ during the swing phase of step i th. $U_{k}\left(i\right)$ represented the controller’s output stimulation intensity to the GAS or TA in the step i th, $U_{k}\left(i-1\right)$ was the controller’s output stimulation intensity to the GAS or TA in the step $\left(i-\right.$ 1)th, $L_{k}$ denoted the learning parameter, the initial intensity of the first step was set to the maximum stimulation intensity $\bar{I_{k}}$ , and $\underline{I_{k}}$ was the minimum stimulation intensity.

### 2.5 Data analysis

A second-order, low-pass Butterworth filter with a cut-off frequency of 15 Hz was applied to filter the kinematic signals. Additionally, based on a three-segment rigid body model of the lower limbs (Kadaba et al., 1990) and the law of cosines, ankle and knee joint angles were obtained. A sixth-order, low-pass Butterworth filter with a cut-off frequency of 10 Hz was used to filter the kinetic signals. The sum of the forces in the anterior direction measured by the four three-dimensional force sensors represented the AGRF (Schmiedmayer and Kastner, 2000). Moreover, the three-dimensional force data obtained in the treadmill’s no-load operation at the corresponding subject’s comfortable speed was subtracted during the calculation process to eliminate interference of the treadmill’s weight and noise. The joint angles and AGRF were calculated and averaged over continuous steps in each experiment and then normalized to a 100% gait cycle.

The Kolmogorov-Smirnov test was utilized to all data to ensure normal distribution. Subsequently, one-way analysis of the variance with repeated measures was conducted, followed by the Bonferroni *post hoc* analysis to compare the effects of different experimental conditions (NS, SA-ILC DS, and SA-ILC DPS) on gait parameters. The significance level was set at 0.05, and when *p* < 0.05, it indicates a statistically significant difference. The statistical analyses were conducted using SPSS 19 (SPSS, Inc., Chicago, IL, United States of America).

## 3 Result

### 3.1 Gait kinematics

**FIGURE 3:**
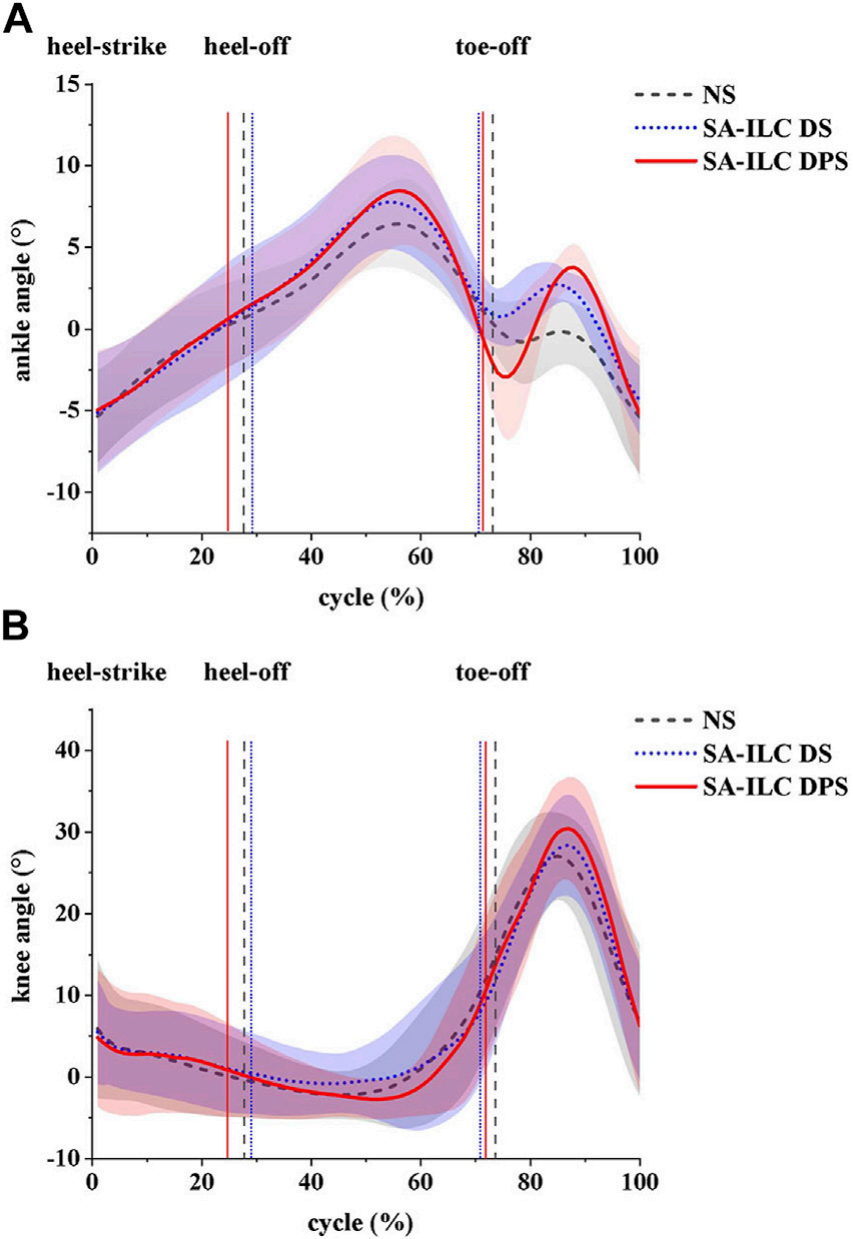
**(A)** ankle angles (mean ± std) and **(B)** knee angles (mean ± std) during the gait cycle for eight post-stroke subjects at comfortable speed.

In this experiment, the normalized ankle and knee angles are illustrated in Figure 3. The lines represents the average angles throughout the gait cycle, while the shaded region denotes the standard deviation corresponding to these angles.

**FIGURE 4:**
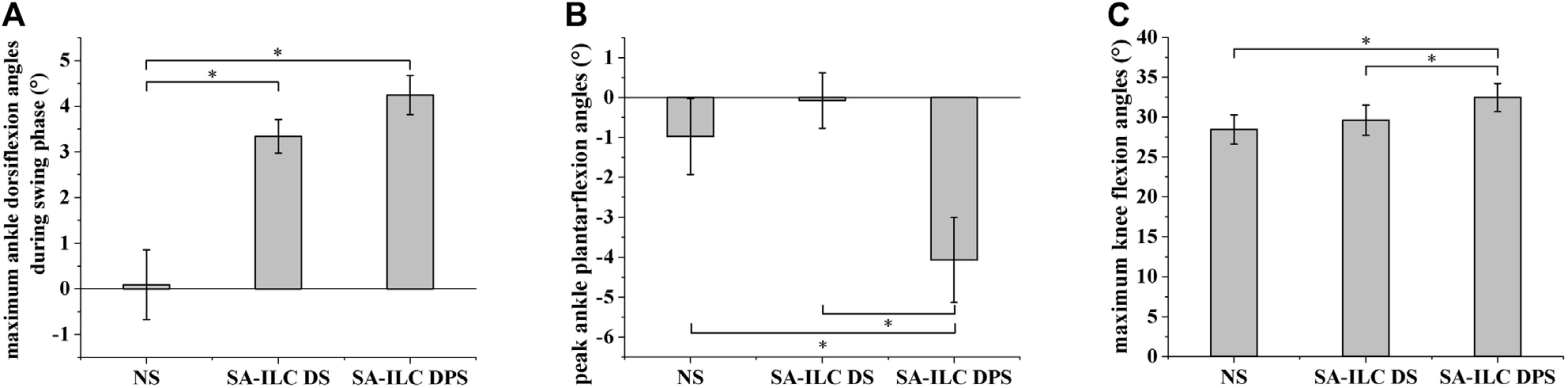
**(A)** Maximum ankle dorsiflexion angles during swing phase for 8 subjects after stroke, *Significant difference (*p* < 0.05). **(B)** Peak ankle plantarflexion angles at toe-off event for 8 subjects after stroke, *Significant difference (*p* < 0.05). **(C)** Maximum knee flexion angles during swing phase for 8 subjects after stroke, *Significant difference (*p* < 0.05).

Figure 4A shows the average maximum ankle dorsiflexion angle during the swing phase of the eight subjects across the three conditions. In the NS, SA-ILC DS, and SA-ILC DPS conditions, the average maximum dorsiflexion angles were 0.09°, 3.34°, and 4.24°, respectively. In contrast to the NS condition, both the SA-ILC DS and SA-ILC DPS conditions exhibited larger ankle dorsiflexion angles (*p* < 0.05). Nevertheless, there was no significant difference between the SA-ILC DS and SA-ILC DPS conditions. In the SA-ILC DS and SA-ILC DPS conditions, the maximum ankle dorsiflexion angles were closer to 4.9° relative to the NS condition.

Figure 4B displays the peak ankle plantarflexion angle of the eight subjects across the three conditions. The peak ankle plantarflexion angles in the NS, SA-ILC DS, and SA-ILC DPS conditions were −0.98°, −0.07°, and −4.07°, respectively. The average peak ankle plantarflexion angle in the SA-ILC DPS condition was significantly larger than those in the NS and SA-ILC DS conditions (*p* < 0.05). No significant difference was observed between the NS and SA-ILC DS conditions.

Figure 4C illustrates the maximum knee flexion angle of the eight subjects across the three conditions. In the NS, SA-ILC DS, and SA-ILC DPS conditions, the maximum knee flexion angles were 28.33°, 29.74°, and 32.45°, respectively. The maximum knee flexion angle in the SA-ILC DS condition closely resembled that observed in the NS condition. However, the maximum knee flexion angle in the SA-ILC DPS condition was significantly larger than those in the NS and SA-ILC DS conditions (*p* < 0.05). There was no significant difference in the maximum knee flexion angle between the SA-ILC DS and NS conditions.

**FIGURE 5:**
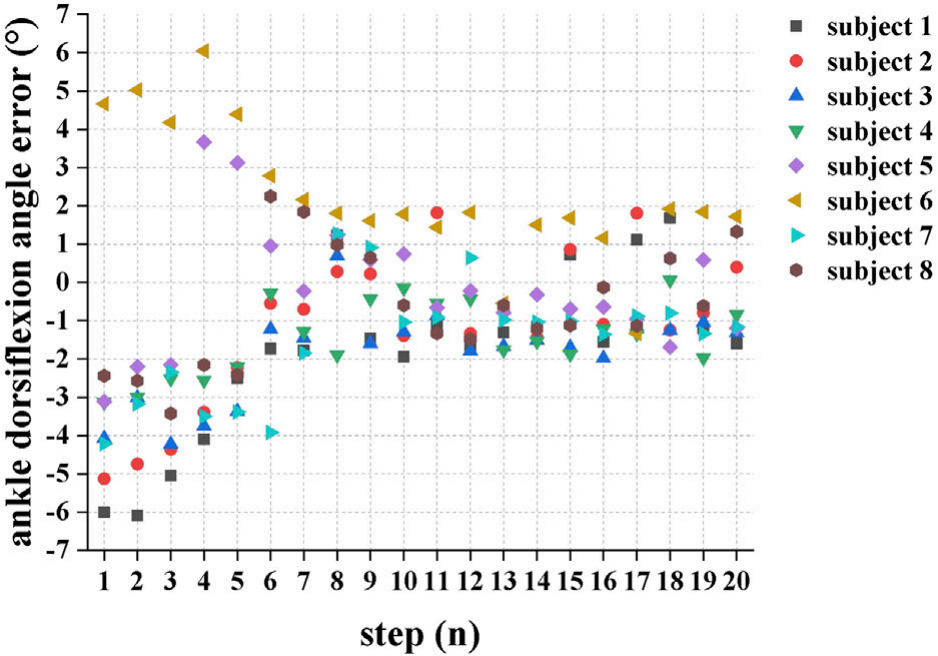
The error between the actual maximum ankle dorsiflexion during swing phase and the target angle of each successive step when the eight subjects performed FES-assisted treadmill walking in the SA-ILC DPS conditions.

Figure 5 demonstrates the maximum ankle dorsiflexion angle error between the actual angle and the target angle during the swing phase for each consecutive step when the eight subjects walked in the SA-ILC DPS condition. Except for subjects 5 and 6, who exhibited larger maximum ankle dorsiflexion angles during the initial few steps, all other participants had ankle dorsiflexion angles smaller than the target angle. At the beginning, the error was relatively substantial, but it diminished to less than 2° after approximately five steps during the gait.

### 3.2 Gait kinetics

**FIGURE 6:**
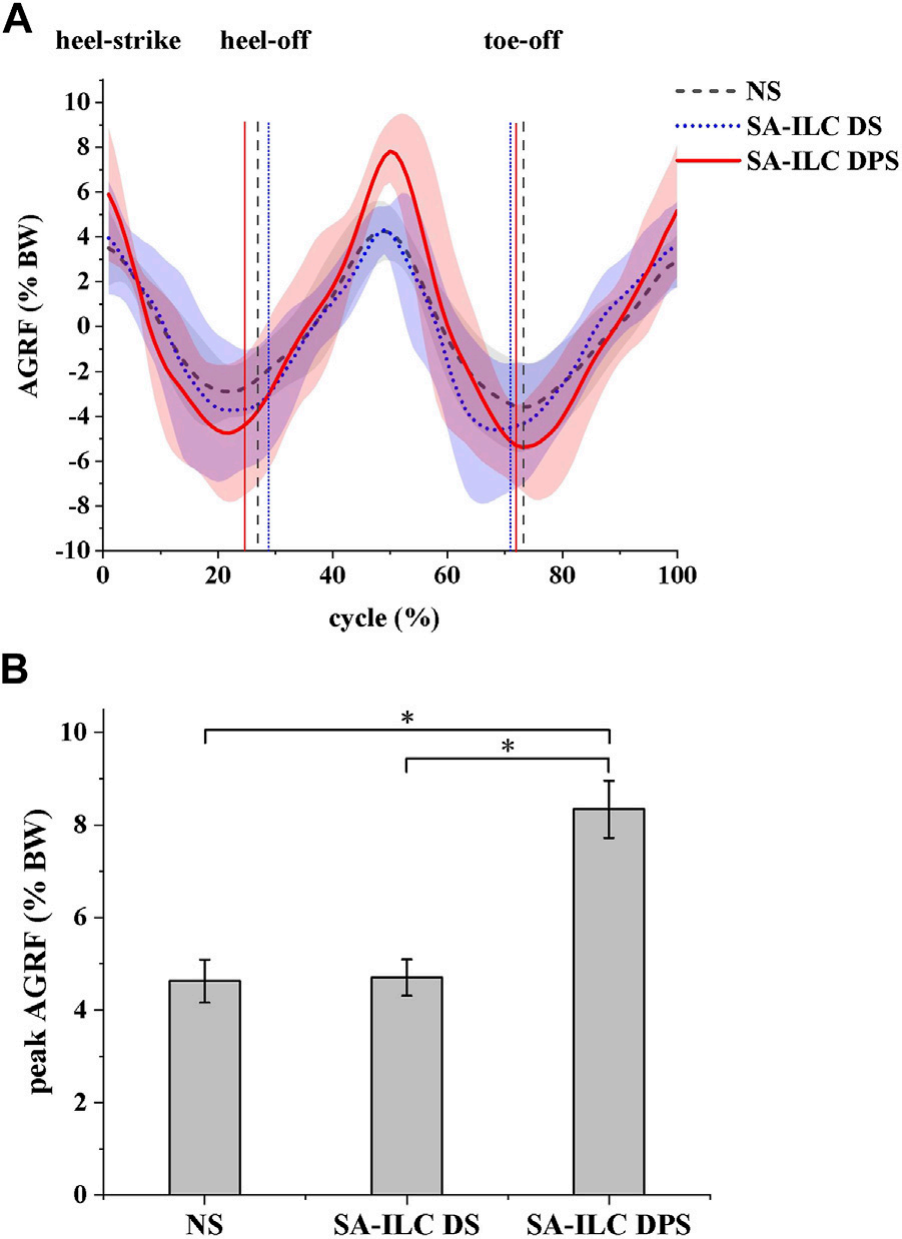
**(A)** The anterior ground reaction force (AGRF) during the gait cycle for eight post-stroke subjects at comfortable speed. **(B)** The peak AGRF during swing phase for 8 subjects after stroke, *Significant difference (*p* < 0.05).

In this experiment, the normalized anterior reaction force (AGRF) is displayed in Figure 6A. The lines and shaded area represent the average AGRF and the corresponding standard deviation throughout the gait cycle, respectively. Figure 6B shows the average peak AGRF during the stance phase, normalized to the subject’s body weight (BW), for the three conditions. The ratios were 4.56%, 4.77%, and 8.34% in the NS, SA-ILC DS, and SA-ILC DPS conditions, respectively. The AGRF percentage relative to body weight in the SA-ILC DPS condition was significantly greater than that in the NS and SA-ILC DS conditions (*p* < 0.05). However, the ratio in the NS condition was similar to that in the SA-ILC DS condition, and there was no significant difference between them.

**FIGURE 7:**
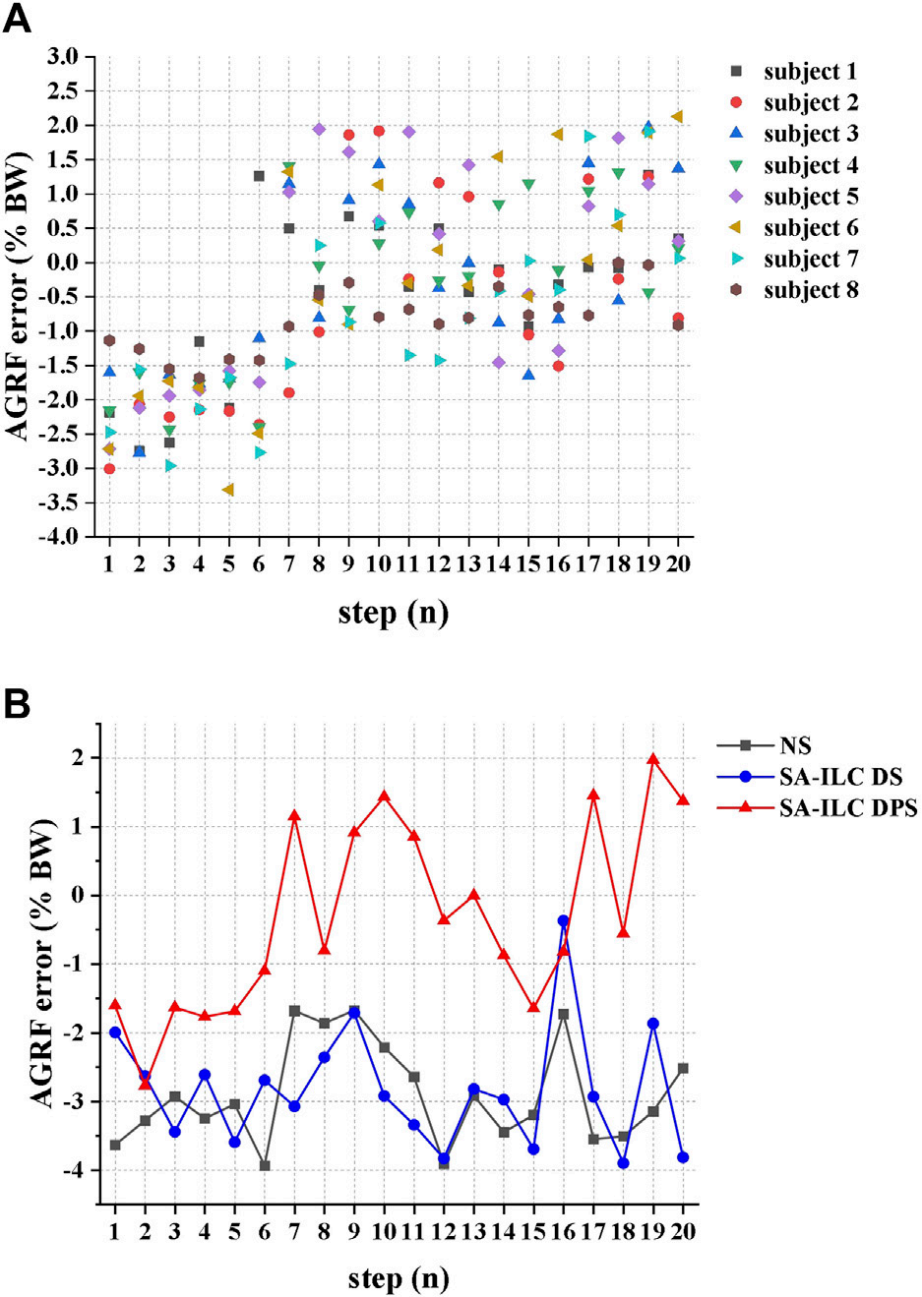
**(A)** The error between the ratio of the peak AGRF during the stance phase to the subject’s body weight and the target ratio of each successive step when 8 subjects performed FES-assisted treadmill walking in the SA-ILC DPS condition. **(B)** The peak AGRF during the stance phase and the target ratio when subject 2 performed FES-assisted treadmill walking in NS, SA-ILC DS, and SA-ILC DPS conditions.

Figure 7A illustrates the error between the ratio of the peak AGRF during the stance phase to the subject’s body weight and the target ratio for each consecutive step when eight subjects walked in the SA-ILC DPS condition. At the beginning of the trial, all subjects displayed relatively large errors, with subject 6 showing the largest error at the fifth step. However, after approximately six steps, the error between the ratio of the average peak AGRF to body weight and the target ratio decreased to a range of −1.5%–2% for all subjects.

Figure 7B displays the error between the ratio of the peak AGRF to the subject’s body weight during the stance phase and the target ratio when subject 2 walked across the three conditions. In the NS, SA-ILC DS, and SA-ILC DPS conditions, the average standard deviations of the errors were −2.90% ± 0.74%, 2.83% ± 0.87%, and −0.33% ± 1.38%, respectively. The mean errors in the SA-ILC DS and SA-ILC DPS conditions were smaller than those in the NS condition (*p* < 0.05).

## 4 Discussion

In this study, a hybrid and adaptive control strategy of FES system was developed for the ankle plantarflexor muscle (GAS) and the dorsiflexor muscle (TA), incorporating a linear model and an ILC controller to respectively modulate stimulation timing and intensity. The orthotic effect of the system for foot drop correction was evaluated by measuring the maximum ankle dorsiflexion angle in the swing phase, peak ankle plantarflexion angle, maximum knee flexion angle in the swing phase and peak AGRF in the stance phase under NS, SA-ILC DS, and SA-ILC DPS conditions.

### 4.1 Timing- and intensity-adaptive control strategy

Triggering stimulation at fixed time intervals, rather than adjusting the stimulation timing according to the walking speed, led to adverse effects such as decreased ankle plantarflexion angle during the push-off phase (Spaich et al., 2014), reduced swing-phase knee flexion angle (Chen et al., 2018b), and diminished forward propulsion (Lee et al., 2014). Furthermore, adaptive adjustment of stimulation intensity was necessary due to the uncertainties in the external environment and disturbances arising from the characteristics of the time-varying muscle (Freeman et al., 2012).

The implemented timing-adaptive linear models of muscle activation/termination time and walking speed were physiologically suitable for FES-assisted gait, which were consistent with the previous research (Chen et al., 2018a; Chen et al., 2018b; Jiang et al., 2020), significantly increasing the swing-phase maximum ankle dorsiflexion angle in patients when compared to the NS condition. Unlike Kesar’s study (Kesar et al., 2009), ankle plantarflexion improved without adversely worsening ankle dorsiflexion in the proposed hybrid and adaptive control strategy, which could be ascribed to optimal stimulation timing. The stimulation timing was adaptively adjusted based on velocity, rather than fixed-phase triggering, which proved to be a more suitable timing for stimulation (Jiang and Song, 2018).

Utilizing the ILC algorithms in the FES system enabled the provision of well-suited stimulation intensity, facilitated swift convergence towards the desired objectives (Seel et al., 2016a) and prevented muscle fatigue. On one hand, the SA-ILC algorithm achieved continuous adaptive modulation of the stimulation intensity for the GAS, allowing patients to reach the most stable AGRF after six steps in gait, with the force error converging to a range of −1.5%–2% BW in subsequent steps. In contrast, the errors in NS and SA-ILC DS conditions were sizable and non-converging. Additionally, this strategy accounted for the relationship between AGRF and walking speed (Ray et al., 2018). By setting different target AGRF values based on the most comfortable walking speed of individual subjects, it achieved personalized adaptive control. On the other hand, the SA-ILC algorithm adaptively modulated the stimulation intensity of the TA, causing the angle error to converge to a range of −2° ∼ 2° after five steps of gait. Conversely, the error in the NS condition was significant and non-converging, consistent with previous results (Jiang et al., 2020). The iterative learning controllers were capable of rapidly converging the error to a minimal range and enhancing the system’s robustness to ensure responsiveness to disturbances arising from internal and external environments (Freeman et al., 2012). Furthermore, prior studies have shown that the ILC strategy could reduce stimulation intensity (Seel et al., 2015), and the SA-ILC strategy was proven to provide adaptive adjustments in stimulation intensity, effectively preventing muscle fatigue (Jiang et al., 2020). It helped avoid fixed or excessive intensity that led to rapid muscle fatigue (Lynch and Popovic, 2008), ensuring improvements in gait kinematics and kinetics.

### 4.2 Hybrid control strategy of FES system

In previous studies, FES was usually applied only to the ankle dorsiflexor (TA) to correct foot drop during the swing phase (Chen et al., 2018b; Jiang et al., 2020). However, merely stimulating the TA was not enough to improve other abnormalities of gait, such as insufficient forward propulsion during the stance phase, and decreased knee flexion angle during the swing phase (Woolley, 2001). The gastrocnemius muscle, serving as both ankle plantarflexor and knee flexor (Wang and Gutierrez-Farewik, 2011), is instrumental in producing push-off forces at terminal stance and raising knee flexion angle (Nadeau et al., 1999; Chen et al., 2005).

The designed hybrid control strategy of FES system not only improved the angles of ankle and knee joints of patients after stroke, but also increased anterior ground reaction force. Significantly, larger ankle dorsiflexion, ankle plantarflexion and knee flexion angles were observed in the SA-ILC DPS condition compared to the NS condition, signifying an enhancement over the results of the dual-channel FES reported in the Kesar study (Kesar et al., 2009). In comparison to NS and SA-ILC DS conditions, the AGRF significantly increased in SA-ILC DPS conditions by over 3% BW, approaching the peak AGRF necessary for ambulating in healthy individuals. The augmentation in AGRF under the SA-ILC DPS condition was not present in the SA-ILC DS. Furthermore, it outperformed the FES system designed by Lee et al. (Lee et al., 2014), which failed to enlarge AGRF. This was attributed to the developed hybrid control strategy of FES that incorporated appropriate stimulation to the GAS. Moreover, the controller utilized peak AGRF as a control parameter, distinguishing it from previous control parameters that only focus on kinematic signals or electromyographic signals. The incorporation of this kinetic parameter effectively enhanced forward propulsion. The study outcomes demonstrated the SA-ILC hybrid system’s ability to rectify insufficient forward propulsion for the paralyzed leg in FES-assisted walking, thereby providing greater kinetic energy for the paretic leg at toe-off and ankle plantarflexion, increasing paretic knee flexion and ankle dorsiflexion in the swing phase, and yielding superior orthotic effects.

### 4.3 Limitations and future work

Despite the numerous advantages of the hybrid timing- and intensity-adaptive control strategy of FES system, the study presented some limitations. Firstly, further research will examine an expanded pool of stroke subjects in longitudinal clinical trials to validate the long-term rehabilitation effects of rehabilitation training. Additionally, only three joint angles and AGRF were utilized to evaluate FES-assisted walking performance. More comprehensive evaluation metrics will be encompassed to optimize control and improve other gait abnormalities, such as center of pressure, gait symmetry, and so on.

## 5 Conclusion

In this study, a hybrid and adaptive control strategy of FES system targeting the tibialis anterior and the gastrocnemius was devised. The system integrated linear models of muscle activation time and walking speed with iterative learning controllers algorithm to respectively modulate stimulation timing and intensity. The proposed SA-ILC hybrid control strategy of FES demonstrated its capacity to enhance maximum ankle dorsiflexion angle, ankle plantarflexion angle and knee flexion angle during the swing phase, as well as anterior ground reaction force. These improvements yielded stable orthotic effects for patients afflicted by foot drop, suggesting promising clinical potential for this innovative approach.

## Data Availability

The raw data supporting the conclusion of this article will be made available by the authors, without undue reservation.
